# Lactylation in urological malignancies: emerging mechanisms and therapeutic direction

**DOI:** 10.3389/fcell.2026.1764134

**Published:** 2026-02-12

**Authors:** Jiafeng Lin, Chenye Yuan, Chunsheng Liu, Minjie Zhang, Jianbin Luo, Zhihuang Wu, Qinghong Ma, Shengbiao Xie, Yufeng Liang, Guoqiang Chen

**Affiliations:** 1 Department of Urology, The Second Hospital of Longyan, Longyan, Fujian, China; 2 The Fourth Clinical College, China Medical University, Shenyang, Liaoning, China; 3 Department of Nephrology, The Second Hospital of Longyan, Longyan, Fujian, China

**Keywords:** bladder cancer, drug resistance, immunity, lactylation, prostate cancer, renal cell carcinoma

## Abstract

Urological malignancies represent a group of highly aggressive tumors with a strong tendency toward therapy resistance. Their pathogenesis is closely associated with metabolic reprogramming and epigenetic regulation. In recent years, lactylation, a novel form of post-translational modification, has garnered significant attention due to its crucial role in linking cellular metabolism with epigenetics. By covalently modifying lysine residues on both histone and non-histone proteins, lactylation dynamically regulates gene transcription and protein function, thereby influencing malignant behaviors in urological malignancies—including proliferation, metastasis, immune evasion, and therapeutic resistance. This review begins by systematically outlining the fundamental characteristics of lactylation and its regulatory networks. It then summarizes the general roles of lactylation in cancer, with a particular emphasis on its mechanisms and functional implications in urological malignancies. Finally, we discuss current research challenges and future directions, aiming to provide new insights into the metabolic–epigenetic interplay in urological malignancies and to establish a theoretical foundation for targeting lactylation as a potential therapeutic strategy and prognostic biomarker.

## 1 Introduction

Urological malignancies, which include bladder cancer (BC), prostate cancer (PCa), and renal cell carcinoma (RCC), are among the most prevalent malignancies worldwide. These cancers pose significant clinical challenges due to their high incidence, frequent recurrence, and therapeutic resistance (Maughan et al., 2025; Barata et al., 2025). Among them, BC stands out as one of the most common urological malignancies, characterized by a high recurrence rate, chemoresistance, and an immunosuppressive tumor microenvironment (TME), all of which contribute to poor patient prognosis (Galsky et al., 2025; Scilipoti et al., 2025; Dyrskjøt et al., 2023). PCa, a leading malignancy in men, frequently develops resistance to hormonal therapies such as enzalutamide and may undergo neuroendocrine differentiation, leading to treatment failure (Nouruzi et al., 2025; Francini et al., 2025). RCC is notably driven by metabolic reprogramming; inactivation of the VHL gene dysregulates hypoxia signaling pathways and enhances the Warburg effect, rendering it largely refractory to conventional radiotherapy and chemotherapy and limiting available treatment options (Larcher et al., 2025; Choueiri et al., 2020; Chappell et al., 2019). Both metabolic reprogramming and epigenetic alterations have been firmly established as central mechanisms in the pathogenesis of urological malignancies (Knowles and Hurst, 2015; Markowski et al., 2019; Davies et al., 2023). In recent years, the convergence of metabolic biology and epigenetics has unveiled lactylation—a novel form of post-translational modification (PTM)—as a key mechanism underlying malignant progression. This emerging field offers novel insights into the pathogenesis of urological malignancies and opens promising avenues for the development of innovative therapeutic strategies (Zhang et al., 2019; Hou et al., 2025).

Lactylation is a PTM in which lactate, a key metabolic intermediate, covalently binds to lysine residues on proteins. This process is dynamically regulated by intracellular lactate levels, lactyltransferases, and delactylases (Sheng X. et al., 2025; Zhang Q. et al., 2025). Initially identified on histones (e.g., H3K18la, H4K12la), lactylation modulates gene transcription by altering chromatin accessibility (Zhang et al., 2019). Subsequent studies have revealed that lactylation also occurs on diverse non-histone proteins—including metabolic enzymes, signaling molecules, and DNA repair factors—where it regulates protein stability, enzymatic activity, and subcellular localization, thereby influencing critical cellular processes such as metabolism, signal transduction, and DNA damage response (Peng and Du, 2025; Li Z. et al., 2025). Moving beyond the conventional view of lactate as merely a metabolic waste product, lactylation represents a fundamental mechanism that bridges cellular metabolism and epigenetic regulation. It serves as a crucial molecular link through which tumor microenvironmental—such as hypoxia and enhanced glycolysis—shape cellular phenotypes in cancer (Rho and Hay, 2025; Llibre et al., 2025).

Recent studies have confirmed the regulatory role of lactylation in urological malignancies, demonstrating tumor-type specificity and complex mechanisms. Although the mechanisms vary, lactylation plays an important role in tumor proliferation, metastasis, and drug resistance. Targeting lactylation may represent a potential therapeutic strategy for urological malignancies. Additionally, prognostic models based on lactation-related genes (LRGs) have shown good predictive value in urological malignancies, effectively predicting patient survival and treatment responses. Although significant progress has been made in lactylation-related research in urological tumors, there remains no targeted systematic review in this field. Thus, there is an urgent need to synthesize existing findings in order to provide direction for future developments. This review first summarizes the classification and regulatory mechanisms of lactylation, then outlines its common roles in cancer. Furthermore, it focuses on lactylation’s functions and mechanisms in various urological malignancies. Finally, we summarize current research challenges and outline future directions, providing theoretical foundations for elucidating metabolic-epigenetic regulatory patterns in urological malignancies and advancing clinical translation research.

## 2 Overview of lactylation

**FIGURE 1:**
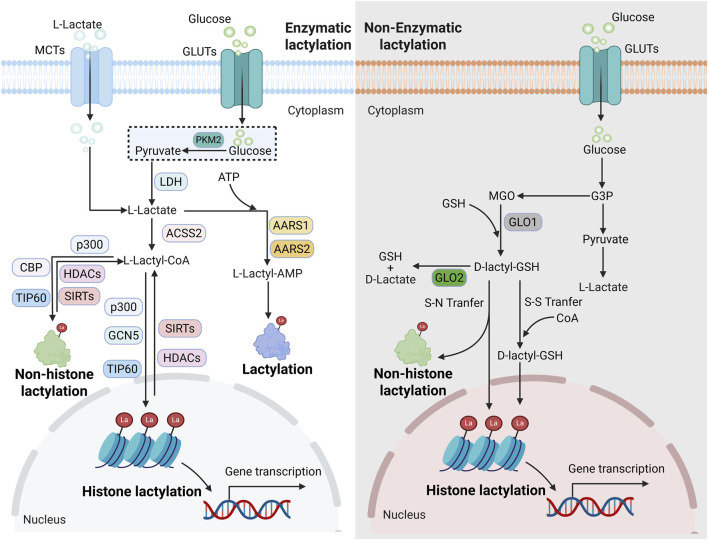
Classification and mechanism of lactylation. Mechanistically, lactylation can be divided into enzyme-catalyzed lactylation and non-enzyme-catalyzed lactylation. In terms of the modified target, lactylation can be divided into histone lactylation and non-histone lactylation.

Lactylation, a novel PTM that has garnered significant attention across various fields in recent years, dynamically regulates gene transcription and protein functions. As an essential initiator of lactylation, lactate is primarily obtained through two pathways within cells: the first involves glucose entering via glucose transporters and producing lactate through glycolysis; the second allows lactate to directly enter cells through receptors like monocarboxylic acid transporters (Chen et al., 2025a; Chen J. et al., 2025; Fan et al., 2025). Upon reaching the cytoplasm, lactate does not bind directly to lysine but must first be converted into L-lactoyl-CoA via L-lactoyl-CoA synthetase—a crucial prerequisite for subsequent binding reactions (Peng and Du, 2025). Due to the presence of two somers (L-and D-lactate), lactylation forms two distinct pathways: L-lactoylation and D-lactoylation (Sheng X. et al., 2025; Iozzo et al., 2025) with the former primarily involving enzymatic lactylation and the latter non-enzymatic lactylation. After binding to lysine, lactate further generates three structural isomers: K_L-la_, K_D-la_, and K_ce_ (Peng and Du, 2025; Zhang D. et al., 2025). This review outlines the classification and regulatory mechanisms of lactylation (Figure 1).

### 2.1 Classification of lactylation

Based on differences in catalytic mechanisms, lactylation can be further categorized into enzymatic and non-enzymatic lactylation (Deng D. et al., 2025). Enzymatic lactylation relies on specific transferases for catalysis, with classical acetyltransferases such as p300 and TIP60 serving as primary mediators (Liu Z. et al., 2025; Yang et al., 2025; Li C. et al., 2025). Studies have confirmed that p300 acts as a core “writer” for lactylation of various histones and non-histone proteins, transferring the lactyl group to target proteins and thereby regulating gene transcription and protein expression (Liu J. et al., 2025; Tang et al., 2025a). In addition, HBO1, GCN5, and MOF have also been identified as writers of lactylation, catalyzing lactylation on a range of histone and non-histone proteins (Zou et al., 2025; Niu et al., 2024; Sun C. et al., 2025). Interestingly, alanyl-tRNA synthetase 1 (AARS1) and AARS2 can directly utilize lactate and ATP to catalyze protein lactylation independently of lactyl-CoA (Li H. et al., 2024). Specifically, AARS1 promotes tumorigenesis by regulating lactylation of p53 (Zong et al., 2024), while AARS2 mediates lactylation of carnitine palmitoyltransferase 2 (CPT2), thereby inhibiting its enzymatic activity and influencing energy metabolism (Mao et al., 2024). In contrast to enzymatic lactylation, evidence for non-enzymatic lactylation remains limited. This process occurs spontaneously under conditions of high lactate concentration or in acidic microenvironments. For instance, in scenarios of glycolytic disruption, D-lactate can undergo non-enzymatic conjugation with lysine residues via lactylglutathione to form K_D-La_ (Gaffney et al., 2020).

Lactylation can be classified into two major categories based on the modified substrate: histone lactylation and non-histone lactylation. Histone lactylation, the first type of lactylation to be discovered, was initially identified in human histones by Zhao et al., in 2019, who detected 28 lactylation sites primarily located on lysine residues of histone H3 and H4 (Zhang et al., 2019). Subsequent studies revealed that additional sites such as H3K9 and H3K14, can also undergo lactylation. Many of these newly identified sites are distributed in promoter or enhancer regions of genes, where they regulate gene transcription and play broad roles in various physiological and pathological processes in humans, particularly in the progression of tumor diseases (Zong et al., 2025; Yan et al., 2025). For example, H4K12la enhances glycolysis in endometrial cells by regulating HIF1-α, thereby improving pregnancy outcomes (Zhao et al., 2023). H3K18la inhibits ferroptosis through the NF-κB pathway, leading to resistance to enzalutamide in PCa (Ji et al., 2025). H3K9la activates SLC7A11 and promotes microglial activation in a mouse model of Parkinson’s disease (Qin et al., 2025). Recent studies have shown that lactylation can also occur on non-histone proteins. Lactylation widely modifies lysine residues on non-histone proteins, thereby influencing protein stability and function, ultimately regulating the progression of various diseases, especially cancers (Chen et al., 2024). For instance, lactylation of p53 contributes to enzalutamide resistance in PCa. Lactylation of RAD51 enhances homologous recombination repair, thereby conferring cisplatin resistance in ovarian cancer (Sun C. et al., 2025). Notably, metabolic enzymes such as pyruvate kinase M2 (PKM2) and enolase can also undergo lactylation, which directly participates in glycolytic reprogramming by altering substrate binding capacity or enzymatic activity (Zong et al., 2025; Wang J. et al., 2022; Shao et al., 2025).

### 2.2 Regulation of lactylation

Lactylation is a dynamic and reversible process. Similar to other PTMs, its regulation relies on the precise collaboration of Writers, Erasers, and Readers (Wang Y. et al., 2025). Writers primarily catalyze lactylation through the catalytic transfer of lactyl groups, with the acetyltransferase family being extensively studied as key contributors. Particularly, p300 plays a central role in regulating lactylation across various histones and non-histone proteins (Liu R. et al., 2025; Fang et al., 2025; Zeng et al., 2023). Additionally, GCN5 promotes myocardial repair gene activation by catalyzing H3K18la (Wang N. et al., 2022). KAT8, which modifies multiple substrates, enhances collagen production to combat skin aging by regulating H4K12la (Zou et al., 2025). AARS1 accelerates endometriosis progression by promoting Snail1 lactylation (Liu L. et al., 2025), while AARS2 inhibits mitochondrial metabolism by modifying CPT2 via racemization (Mao et al., 2024). Furthermore, studies have identified YiaC, HBO1, GCN5, GNAT13, HDAC6, and others as Contributors to racemization (Peng and Du, 2025; Niu et al., 2024; Dong et al., 2022; Sun W. et al., 2023; Li et al., 2023; Sun et al., 2024).

Erasers function by removing lactyl groups, with HDACs serving as the primary family of delactylases. For example, HDAC1 can reverse ASH2L lactylation, thereby inhibiting angiogenesis and malignant progression in hepatocellular carcinoma (HCC) (Han et al., 2025). Similarly, HDAC2 also suppresses angiogenesis by reducing H3K9la (Fan et al., 2024). The sirtuin family exerts delactylation activity in an NAD^+^-dependent manner. For instance, SIRT1 reverses HADHA lactylation and ameliorates sepsis-induced cardiac dysfunction (Zhang TN. et al., 2025). SIRT2 inhibits cuproptosis in gastric cancer by suppressing methyltransferase 16 (METTL16) lactylation (Sun L. et al., 2023). Additionally, CobB has been identified as a novel lysine lactylation eraser that modulates PykF activity by removing its lactylation modification (Dong et al., 2022). Furthermore, HDAC3 and SIRT3 have also been recognized as erasers of lactylation (Sun M. et al., 2025; Chen et al., 2025c).

Readers function by recognizing lactylation. Currently, the only identified Readers are DPF2, Brg1, and TRIM33. Among these, TRIM33 represents a novel histone lactylation reader that regulates the expression of inflammatory genes in activated macrophages and mediates the polarization process of M2-type macrophages (Nuñez et al., 2024). DPF2 promotes tumorigenesis by reading lactylation of H3K14 and driving the transcription of cancer-related genes (Zhai et al., 2024). Brg1 facilitates mesenchymal–epithelial transition in pluripotent stem cells by recognizing lactylation signals at H3K18 (Hu et al., 2024). The identification of these readers provides critical insights into how lactylation precisely regulates gene expression.

## 3 The role of lactylation in cancer

**FIGURE 2:**
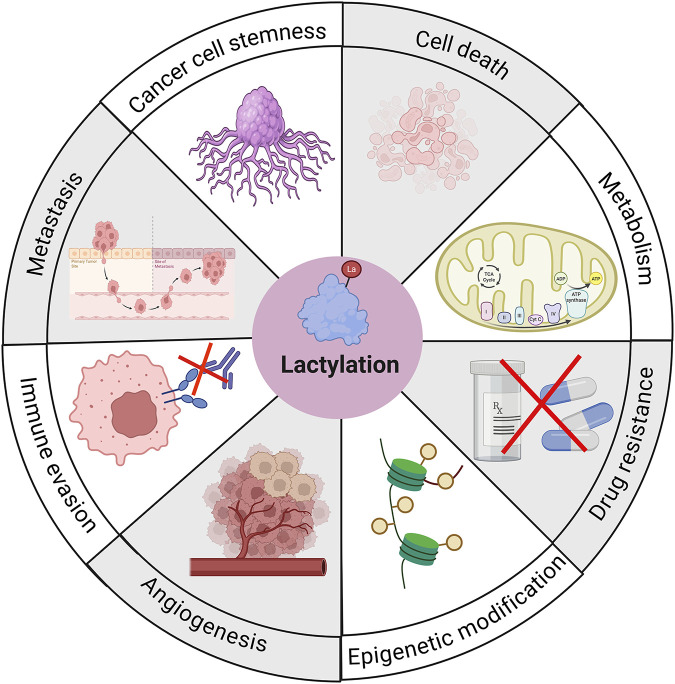
The role of lactylation in cancer. Lactylation regulates multiple biological processes, including metabolic reprogramming, epigenetics, cell death, angiogenesis, cancer metastasis, drug resistance, cancer stemness, and immune evasion, ultimately driving cancer progression.

In recent years, the critical role of lactylation in cancer has been increasingly recognized. Through various complex mechanisms, lactylation regulates multiple biological processes including metabolic reprogramming, epigenetics, cell death, angiogenesis, cancer metastasis, drug resistance, cancer stemness, and immune evasion, ultimately driving cancer progression (Figure 2) (Rho and Hay, 2025; Lv et al., 2025; Wang D. et al., 2025).

### 3.1 Lactylation and cancer metabolic reprogramming

Metabolic reprogramming is a hallmark of cancer. Enhanced glycolysis driven by metabolic reprogramming leads to increased lactate production, which in turn promotes lactylation. Conversely, lactylation can further amplify glycolysis through various metabolic pathways, forming a positive feedback loop that facilitates cancer progression (Hou et al., 2025; Brown and Ganapathy, 2020). For example, in pancreatic cancer, Ras homolog family member F upregulates c-Myc expression, promotes PKM2 transcription, and enhances glycolysis and lactate accumulation. The accumulated lactate then promotes epithelial–mesenchymal transition (EMT) in pancreatic cancer by inducing lactylation and nuclear translocation of Snail1 (Zhao R. et al., 2024). In HCC, lactylation of c-Myc enhances its stability, thereby increasing glycolytic activity in cancer cells (Yao and Yang, 2024). Furthermore, lactylation directly regulates the expression of metabolic genes, further promoting metabolic reprogramming in the TME (Li M. et al., 2024; Tian et al., 2024). This bidirectional regulatory relationship ultimately drives tumor progression and also offers potential targets for cancer therapy.

### 3.2 Lactylation and cancer epigenetic regulation

As a novel form of PTM, the role of lactylation in cancer epigenetics has become increasingly clear in recent years, providing new perspectives for deciphering the molecular mechanisms of cancer. Lactate accumulated in cancer cells can promote protein lactylation through enzymatic or non-enzymatic pathways, thereby participating in epigenetic regulation. For example, lactylation at sites such as H3K18 and H4K12 can influence cancer progression by regulating the transcription of related genes (Hu et al., 2024; Deng J. et al., 2025). Lactylation of β-catenin and p53 affects tumorigenesis and stemness by modulating their stability and function (Zong et al., 2024; Miao et al., 2023). Meanwhile, lactylation engages in crosstalk with classical modifications such as acetylation and phosphorylation, thereby regulating cancer progression through complex mechanisms (Li et al., 2025c; Duan et al., 2024). Lactylation and epigenetics in cancers represent a major direction for future research.

### 3.3 Lactylation and cancer cell death

Cancer cells often exhibit resistance to programmed cell death, a key mechanism underlying their survival and drug tolerance. Lactylation participates in regulating cancer cell death resistance through multiple pathways. Firstly, lactylation enhances cancer cell survival by stabilizing anti-apoptotic proteins or inhibiting pro-apoptotic signaling. For example, in melanoma, H3K18la suppresses cancer cell apoptosis by accelerating the degradation of tumor suppressor genes PER1 and TP53 mRNA (Yu et al., 2021). Meanwhile, lactylation can influence tumor cell survival by modifying key autophagy-related proteins. For instance, H3K18la promotes the expression of the autophagy-enhancing protein RUBCNL, leading to resistance to bevacizumab therapy in colorectal cancer (CRC) (Li W. et al., 2024). Additionally, lactylation modulates ferroptosis to affect cancer progression. As an example, lactylation of NSUN2 enhances the activity of glutamate–cysteine ligase catalytic subunit, induces elevated glutathione synthesis, and ultimately confers resistance to ferroptosis in cancer cells (Niu et al., 2025). These findings suggest that targeting lactylation may reverse cancer cell resistance to cell death, thereby improving the efficacy of existing cancer treatments.

### 3.4 Lactylation and cancer angiogenesis

Cancer angiogenesis is a critical process through which cancers obtain nutrients and oxygen to support growth and metastasis. Lactylation plays a key regulatory role in cancer-associated angiogenesis. Studies have shown that lactylation promotes angiogenesis in HCC by regulating Golgi phosphoprotein 73 (Ye et al., 2024). In glioblastoma, lactylation has also been found to promote vasculogenic mimicry development (Zhang et al., 2023). Furthermore, the role of lactylation in regulating angiogenesis was recently confirmed in PCa, where targeting lactylation significantly suppressed angiogenesis (Yu et al., 2023; Luo et al., 2022). Although the mechanisms by which lactylation regulates cancer angiogenesis require further investigation, it has emerged as a potential target for anti-angiogenic therapy, offering new directions for combination treatment strategies.

### 3.5 Lactylation and cancer metastasis

Metastasis is a major cause of cancer-related mortality, and lactylation plays a regulatory role in multiple key steps of cancer metastasis. Specifically, in gastric cancer, H3K18la upregulates the expression of Vascular Cell Adhesion Molecule 1, thereby activating the AKT-mTOR signaling pathway and enhancing the invasive ability of cancer cells (Zhao Y. et al., 2024). In CRC, H3K9la promotes metastasis by upregulating GRAMD1A expression and modulating cholesterol metabolism (Zhang C. et al., 2025). In pancreatic cancer, lactylation of Snail1 protein facilitates EMT, thereby promoting cancer cell migration and invasion (Zhao R. et al., 2024). These findings suggest that lactylation may serve as a potential target for intervening in cancer metastasis.

### 3.6 Lactylation and cancer drug resistance

Drug resistance is a major cause of cancer treatment failure. Lactylation plays an important role in various drug resistance processes, offering new directions to overcome therapeutic limitations. Firstly, lactylation can influence chemotherapy resistance in multiple cancers. For example, in CRC, lactylation enhances the stability of CEACAM6, leading to tumor cell resistance to 5-fluorouracil (Chu et al., 2023). In breast cancer, histone lactylation upregulates zinc finger protein 64 expression, resulting in doxorubicin resistance (Zhang K. et al., 2025). Additionally, recent studies have also reported associations between lactylation and resistance to immunotherapy as well as targeted therapy (Deng D. et al., 2025; Zhu et al., 2025; Chen Q. et al., 2025). These findings suggest that targeting lactylation may reverse cancer treatment resistance and improve therapeutic efficacy.

### 3.7 Lactylation and cancer stemness

Cancer stemness is a core property that enables tumor cells to maintain self-renewal capacity, differentiation potential, and drug resistance. Lactylation contributes to the maintenance of cancer stem cell stemness by regulating the transcription of key genes and protein expression. Specifically, in CRC, hypoxia-induced lactylation of β-catenin activates the Wnt pathway, thereby enhancing cancer cell stemness (Miao et al., 2023). In breast cancer, lactylation of ZMIZ1 influences cancer cell stemness by increasing the transcriptional activity of Nanog (Liu Y. et al., 2025). Additionally, lactylation of polypyrimidine tract-binding protein 1 enhances the expression of the glycolytic enzyme PFKFB4, sustaining the stem-like properties of glioma stem cells (Zhou et al., 2025). Collectively, these findings suggest that targeting lactylation may represent a key strategy for reversing cancer stemness and reducing malignant potential.

### 3.8 Lactylation and cancer immune evasion

Immune evasion is the core strategy enabling cancers to evade immune surveillance and achieve malignant progression, with lactylation playing a pivotal role in regulating this process. lactylation not only directly affects cancer cells but also reshapes immune cell functions, thereby promoting immune evasion. Studies have shown that lactylation can induce macrophages to polarize into tumor-promoting M2 cells. For instance, in glioblastoma, H3K18la upregulates the expression of tumor necrosis factor superfamily member 9, which induces M2 macrophage polarization (Li M. et al., 2024). Additionally, in CRC, H3K9la activates METTL1 transcription, leading to elevated expression of the immune checkpoint molecule CD155. The high expression of CD155 suppresses the activity of natural killer cells and CD8^+^ T cells, thereby exacerbating cancer immune evasion (Wang F. et al., 2024). Furthermore, lactic acidified MOESIN enhances the activity of the TGF-β signaling pathway, promoting the generation of regulatory T cells and further suppressing anti-tumor immune responses (Gu et al., 2022). These findings indicate that lactylation plays a critical role in constructing an immunosuppressive TME. Targeting lactylation may enhance the response rate of immunotherapy.

## 4 The role and mechanism of lactylation in BC

**FIGURE 3:**
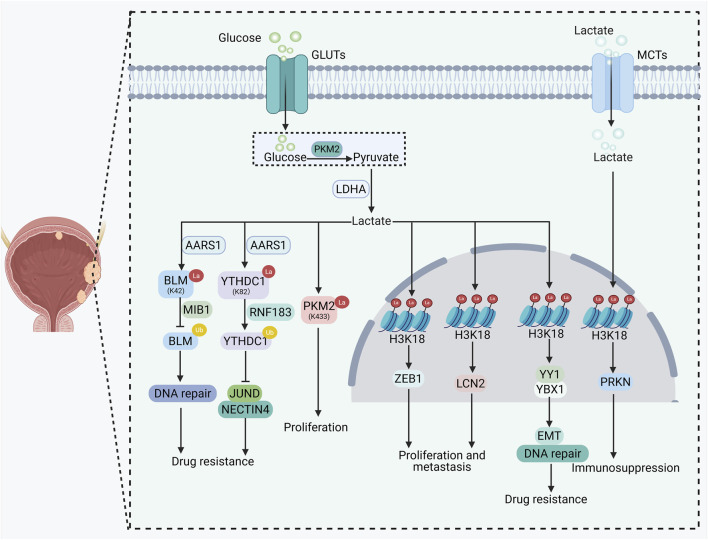
The role and mechanism of lactylation in bladder cancer.

Recent studies have progressively revealed the crucial role of lactylation in BC progression (Table 1; Figure 3). Among these, histone lactylation is extensively involved in the progression, drug resistance, and immune evasion of BC by modulating the transcriptional activity of specific genes. Phosphofructokinase-1 (PFK-1) enhances glycolysis and lactate production, leading to increased H3K18la levels, which in turn activate zinc finger E-box-binding homeobox 1 (ZEB1) transcription and promote the proliferation, migration, and invasion of BC cells. Knockdown of PFK-1 suppresses this process by reducing H3K18la (Wang R. et al., 2024). Similarly, circXRN2 inhibits SPOP-mediated degradation of LATS1 by activating the Hippo pathway, thereby decreasing H3K18la levels and suppressing the expression of the oncogene LCN2, ultimately inhibiting tumor progression (Xie et al., 2023). Histone lactylation is also closely associated with drug resistance in BC. H3K18la activates the transcription of YY1 and YBX1 by enriching at their promoter regions, promoting DNA damage repair and EMT, ultimately leading to cisplatin resistance. Inhibition of H3K18la restores cisplatin sensitivity (Li F. et al., 2024). Furthermore, the hypoxic TME upregulates LDHA to enhance lactate production, which subsequently catalyzes H3K18la. This modification activates RBM15 transcription, leading to elevated IGFBP3 protein expression. The upregulated IGFBP3 interacts with phosphorylated EGFR and DNA-PKcs, facilitating their nuclear translocation and activating the non-homologous end joining repair pathway, ultimately conferring cisplatin resistance in BC cells (Sun J. et al., 2025). Furthermore, histone lactylation can regulate the immune microenvironment of BC. Studies have shown that H3K18la directly upregulates PRKN expression, enhances mitophagy, promotes M2-type tumor-associated macrophage (TAM) polarization, and fosters an immunosuppressive microenvironment, thereby facilitating immune escape of BC cells (Deng X. et al., 2025). Similarly, LDHA-mediated lactate production promotes H4K5la in BC cells in a manner dependent on EP300. This modification directly binds to the promoter region of PD-L1 and activates its transcription, thereby enhancing the immune evasion capacity of tumor cells (Tang et al., 2026). In addition, loss of the histone demethylase KDM6A impairs glycolysis and reduces lactate output in BC cells, which subsequently decreases histone lactylation levels—including H3K9la and H3K18la—in Tregs. This downregulates the expression of key immunosuppressive genes such as Foxp3, Tgfb, and PD-1, ultimately attenuating the immunosuppressive function of Tregs (Singh et al., 2026).

**TABLE 1:** The role and mechanism of lactylation in urologic malignancies.

Cancer	Substrate	Site	Mechanism	Outcome	References
BC	H3	K18	PFK-1 promotes glycolysis and lactate production, which in turn enhances H3K18la to activate the transcriptional activity of ZEB1.	Promotes proliferation and metastasis	Wang et al. (2024b)
	H3	K18	H3K18la drives malignant phenotypes by promoting the transcription of LCN2.	Promotes proliferation and metastasis	Xie et al. (2023)
	H3	K18	H3K18la promotes the activation of drug resistance-related pathways by upregulating the expression of YY1 and YBX1.	Promotes drug resistance	Li et al. (2024d)
	H3	K18	H3K18la activates RBM15 transcription to elevate IGFBP3; IGFBP3 binds to phosphorylated EGFR and DNA-PKcs, promoting their nuclear translocation and enhancing DNA repair.	Promotes drug resistance	Sun et al. (2025c)
	H3	K18	H3K18la upregulates PRKN expression to enhance mitophagy activity, thereby promoting M2 macrophage polarization and fostering an immunosuppressive microenvironment.	Promotes immunosuppression	Deng et al. (2025c)
	H4	K5	LDHA-mediated lactate production promotes H4K5la, which in turn drives the transcriptional activation of PD-L1 in BC cells.	Promotes immune evasion	Tang et al. (2026)
	H3	K9, K18	Downregulates the expression of key immunosuppressive genes, such as Foxp3, Tgfb, and PD-1.	Promotes immune evasion	Singh et al. (2026)
	HNRNPA1	K350	Lactate derived from glycolysis promotes HNRNPA1la, which drives PKM2 to shift into a pro-glycolytic isoform, thereby establishing a “glycolysis–HNRNPA1 lactylation–PKM2” positive feedback loop.	Promotes proliferation and metastasis	Wang et al. (2025c)
	BLM	K24	AARS1-mediated lactylation of BLM suppresses its ubiquitination and degradation by MIB1, while promoting its interaction with DNA repair factors, thereby accelerating DNA repair.	Promotes drug resistance	Li et al. (2025d)
	YTHDC1	K82	Lactylation of YTHDC1 promotes its ubiquitination and degradation by RNF183. The decrease in YTHDC1 suppresses NECTIN4 expression by reducing the stability of JUND.	Promotes drug resistance	Xing et al. (2025)
	PKM2	K433	Lactylation of PKM2 maintains its cytoplasmic localization and glycolytic activity.	Promotes tumor proliferation	Jin et al. (2025)
	DHX15	K17	HDAC2 interacts with DHX15 and removes its lactylation, thereby altering the alternative splicing of the downstream ribosomal protein RPL9.	Inhibits proliferation, metastasis and drug resistance	Xu et al. (2025)
PCa	H3	K18	p300-mediated H3K18la activates the NF-κB pathway to upregulate RPS6KC1 expression, thereby reducing lipid reactive oxygen species accumulation and inhibiting ferroptosis.	Promoting drug resistance	Ji et al. (2025)
	p53	-	SLC4A4 enhances glycolysis by activating the NF-κB/STAT3 axis, the resulting lactate accumulation induces p53 lactylation and suppresses its function.	Promotes drug resistance	Chen et al. (2024)
	H3	K18	TOP2A upregulates LDHA to enhance glycolysis and lactate production, lactate, in turn, promotes TOP2A transcription via H3K18la, forming a vicious cycle.	Promotes proliferation and metastasis	Tian et al. (2024)
	H3	K18	H3K18la promotes PD-L1 expression and suppresses Sema3A by enhancing HIF1A transcriptional activity.	Promotes angiogenesis and immune evasion.	Yu et al. (2023)
	HIF1α	-	Lactylation of HIF1α upregulates the expression of KIAA1199, which activates the VEGFA pathway and suppresses sema3A, thereby promoting angiogenesis.	Promoting invasion and drug resistance	Luo et al. (2022)
	H3	K18	ZEB1 upregulates glycolytic enzymes to promote lactate accumulation, which in turn enhances H3K18la and drives the transdifferentiation of PCa toward a neuroendocrine phenotype.	Promotes neuroendocrine transdifferentiation	Wang et al. (2024c)
	H3	K18	Dysfunction of the Numb/Parkin axis leads to enhanced glycolysis and lactate accumulation, which upregulates H3K18la and activates the transcription of neuroendocrine-related genes.	Promotes neuroendocrine transdifferentiation	He et al. (2023)
	H3	K18	H3K18la upregulates LDHA by activating the expression of LHX2, forming a glycolysis–lactylation–LHX2 positive feedback loop that ultimately elevates DNMT1.	Promotes neuroendocrine transdifferentiation	Jiang et al. (2025)
	H3	K18	H3K18la activates FOXM1 transcription.	Promotes proliferation	Sheng et al. (2025b)
	H3	K18	PTEN/p53 deficiency activates the PI3K/AKT and Wnt/β-catenin pathways, enhances glycolysis, and leads to lactate accumulation. Lactate, in turn, suppresses phagocytic function and maintains the immunosuppressive phenotype of TAMs by inducing H3K18la.	Promotes immune evasion	Chaudagar et al. (2023a)
	H3	K18	Hyperactivation of the PI3K pathway promotes lactate production. The accumulated lactate suppresses the phagocytic function of TAMs via H3K18la.	Promotes immune evasion	Chaudagar et al. (2023b)
	CNPY3	-	Lactylation of CNPY3 inhibits pyroptosis via the caspase-1/GSDMD pathway.	Inhibits pyrolysis	Zhang et al. (2024)
RCC	H3	K18	H3K18la activates the transcription of PDGFRβ, which in turn further enhances H3K18la by promoting glycolysis and lactate production, forming a positive feedback loop.	Promotes proliferation and metastasis	Yang et al. (2022)
	H3	K18	L-2-HG enhances HIF1A transcription through promoting H3K18la, which in turn inhibits ferroptosis in tumor cells.	Promotes proliferation and metastasis	Liu et al. (2025g)
	H3	K14, K18, K56	FKBP10 enhances the activity of LDHA and lactate production by promoting its phosphorylation. Lactate, in turn, regulates the expression of metabolism-related genes through histone lactylation.	Promotes proliferation and metastasis	Liu et al. (2024)
	H3	K18	NSUN2 enhances glycolysis and elevates H3K18la levels by stabilizing NEO1 mRNA, subsequently, H3K18la upregulates PD-L1 through the MYC/POM121/CD274 axis.	Promotes proliferation, metastasis, and immune evasion	Wang et al. (2025e)
	YTHDC1	K82	The hypoxic microenvironment induces p300-mediated lactylation of YTHDC1, which protects BCL2 and E2F2 mRNAs from degradation mediated by the PAXT complex.	Promotes survival and proliferation	Dai et al. (2025)
	MDH2	K239	Lactylation of MDH2 enhances its enzymatic activity and elevates NADPH levels through the MDH2/CS/SLC25A1 complex, thereby helping tumor cells resist oxidative stress and maintain mitochondrial function.	Promotes proliferation and metastasis	Tang et al. (2025b)

Abbreviations: BC, bladder cancer; PFK-1, phosphofructokinase-1; ZEB1, zinc finger E-box-binding homeobox 1; AARS1, alanyl-tRNA, synthetase 1; PKM2, pyruvate kinase M2; TAMs, tumor-associated macrophages; CNPY3, canopy FGF, signaling regulator 3; PDGFRβ, platelet-derived growth factor receptor β; MDH2, malate dehydrogenase 2.

Non-histone lactylation also plays a pivotal role in multiple malignant processes of BC, including drug resistance and immune regulation. In BC, lactate derived from excessive glycolysis promotes the lactylation of the RNA-binding protein HNRNPA1 via catalysis by P300. This modification enhances the ability of HNRNPA1 to regulate the alternative splicing of PKM pre-mRNA, favoring the production of the pro-glycolytic PKM2 isoform and thereby establishing a “glycolysis–HNRNPA1 lactylation–PKM2” positive-feedback loop. This loop continuously drives tumor cell proliferation, migration, and invasion (Wang T. et al., 2025). It contributes to chemoresistance through multiple mechanisms. On one hand, lactate derived from glycolysis is catalyzed by AARS1 to promote lactylation of BLM, a key helicase in homologous recombination repair. This modification enhances BLM stability by inhibiting MIB1-mediated ubiquitination degradation, facilitates its interaction with DNA2 and TOPIIA, accelerates DNA repair, and leads to resistance to anthracycline drugs. Irinotecan can target this process to reverse drug resistance (Li et al., 2025d). On the other hand, excessive lactate produced in a high-glucose TME activates AARS1-mediated lactylation of YTHDC1, enhancing RNF183-dependent ubiquitination and degradation of YTHDC1. This reduces JUND mRNA stability in an m6A-dependent manner, suppresses NECTIN4 expression, and decreases sensitivity to enfortumab vedotin (Xing et al., 2025). Non-histone lactylation is also involved in immune regulation in BC through diverse mechanisms. Research by Jin et al. demonstrated that lactylation of PKM2 maintains its cytoplasmic localization and glycolytic activity, promoting proliferation. Mannose inhibits PKM2 enzymatic activity, reduces lactate production, decreases its lactylation, and promotes acetylation. Acetylated PKM2 translocates into the nucleus, activates the NF-κB pathway, induces pyroptosis, and enhances immune responses (Jin et al., 2025). Additionally, FASN and RUNX2 indirectly enhance non-histone lactylation by promoting lactate production, driving tumor proliferation, modulating immune cell infiltration, and contributing to immune evasion. Their high expression is associated with poor prognosis, though the specific proteins lactylated by this mechanism require further identification (Chen Y. et al., 2025). Lactylation can exert not only pro-tumorigenic but also tumor-suppressive effects in BC. Study has reported that overexpression of HDAC2 in BC significantly reduces pan-lactylation levels, particularly the lactylation of the spliceosome pathway protein DHX15. This alteration subsequently modulates the alternative splicing of the downstream ribosomal protein RPL9, ultimately promoting BC cell proliferation, migration, stemness, and cisplatin resistance (Xu et al., 2025). These findings suggest a dual regulatory role of lactylation in BC, although further research is needed to substantiate this perspective.

Furthermore, models constructed based on LRGs demonstrate strong predictive capability for prognosis and treatment response in BC. One study found that a gene set comprising specific LRGs could predict the survival of BC patients, with high lactylation scores associated with poorer prognosis but potentially greater sensitivity to immune checkpoint inhibitors (Liu et al., 2025f). Another study developed a model consisting of 12 LRGs that effectively distinguished between high-risk and low-risk BC patients. High-risk patients exhibited active glycolysis and hypoxia pathways and were more sensitive to chemotherapy, whereas low-risk patients showed better responses to immunotherapy (He et al., 2024). Similarly, Zhao et al. constructed a prognostic model based on LRGs using transcriptomic and single-cell sequencing data, which effectively predicted both survival and immunotherapy efficacy in BC patients (Zhao et al., 2025). Additionally, models built around LRGs such as FASN and RUNX2 indicated that high expression of these genes is linked to unfavorable prognosis in BC. These genes influence tumor progression by regulating lactylation, providing valuable references for prognostic assessment and treatment selection (Chen Y. et al., 2025).

## 5 The role and mechanism of lactylation in PCa

**FIGURE 4:**
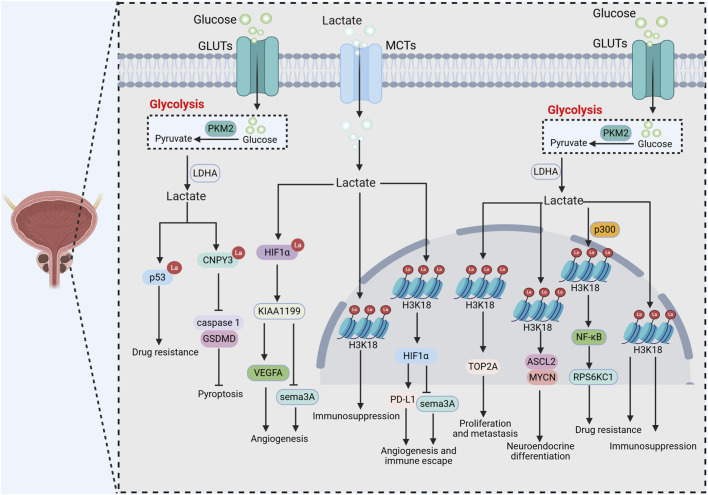
The role and mechanism of lactylation in prostate cancer.

The role of lactylation in PCa has recently been increasingly elucidated (Table 1; Figure 4). Several studies have revealed that histone lactylation plays a critical role in the progression, drug resistance, and immune microenvironment remodeling of PCa. Specifically, the environmental pollutant BDE-47 activates a TOP2A/LDHA positive feedback loop: TOP2A upregulates LDHA to enhance glycolysis and lactate production, while lactate further promotes TOP2A transcription via H3K18la, forming a vicious cycle that drives PCa cell proliferation (Tian et al., 2024). Additionally, ZEB1 transcriptionally upregulates glycolytic enzymes such as HK2 and LDHA, promoting lactate accumulation and subsequent H3K18la formation. This activates neuroendocrine-related genes, including Mycn and Ascl1, facilitating the transdifferentiation of prostate adenocarcinoma into a neuroendocrine phenotype (Wang D. et al., 2024). Similarly, Numb/Parkin-mediated mitochondrial quality control defects lead to metabolic reprogramming, and lactate accumulation-induced H3K18la upregulates neuroendocrine-associated genes, promoting the development of therapy-resistant phenotypes of PCa (He et al., 2023). Furthermore, long-term androgen deprivation therapy induces glycolysis and histone lactylation (particularly H3K18la), which upregulates LDHA by activating the transcription factor LHX2. This establishes a glycolysis–lactylation–LHX2 positive feedback loop, ultimately promoting neuroendocrine differentiation of PCa through the upregulation of DNMT1 (Jiang et al., 2025). Histone lactylation is also closely associated with PCa drug resistance. Study has revealed that p300-mediated H3K18la activates the NF-κB pathway, upregulating the expression of RPS6KC1, which in turn reduces lipid reactive oxygen species accumulation and inhibits ferroptosis, ultimately leading to the development of enzalutamide resistance in PCa (Ji et al., 2025). H3K18la also promotes PCa proliferation, inhibits apoptosis, and enhances lactate metabolic activity by activating FOXM1 transcription, ultimately contributing to chemotherapy resistance (Sheng W. et al., 2025). Within the immune microenvironment, PTEN/p53-deficient tumor cells enhance glycolysis through the PI3K/AKT pathway and Wnt pathway. Lactate derived from glycolysis induces H3K18la in TAMs, suppressing their phagocytic function and maintaining an immunosuppressive phenotype. Combined inhibition of PI3K, MEK, and Wnt pathways reduces H3K18la levels and restores TAM activity to control tumor growth (Chaudagar et al., 2023a; Chaudagar et al., 2023b). Furthermore, lactate in the TME enhances HIF1A transcriptional activity via H3K18la, promotes PD-L1 expression, and inhibits the anti-angiogenic factor Sema3A, exacerbating angiogenesis and immune escape (Yu et al., 2023).

Non-histone lactylation has recently been confirmed to play a role in PCa, closely associated with drug resistance, angiogenesis, and regulation of cell death processes. Firstly, non-histone lactylation contributes to drug resistance in PCa. Studies have shown that SLC4A4 enhances glycolysis by activating the NF-κB/STAT3 axis, and the resulting lactate accumulation induces lactylation of p53, inhibiting its function and leading to resistance to enzalutamide. Knockdown of SLC4A4 reverses this process (Chen et al., 2024). Secondly, in terms of angiogenesis, lactate enters cells via MCT1 and induces lactylation of HIF1α, stabilizing it under normoxic conditions and upregulating KIAA1199 expression. KIAA1199 promotes angiogenesis by activating the VEGFA signaling pathway while suppressing the anti-angiogenic molecule sema3A, ultimately enhancing tumor invasiveness and therapy resistance (Luo et al., 2022). Lastly, non-histone lactylation also regulates pyroptosis in PCa. Gambogic acid recruits the delactylase SIRT1 to remove lactylation from canopy FGF signaling regulator 3 (CNPY3), resulting in altered CNPY3 localization and lysosomal rupture, which ultimately induces pyroptosis through the caspase 1/GSDMD pathway (Zhang et al., 2024).

Models constructed based on LRGs have also demonstrated considerable potential in prognostic prediction for PCa. A prognostic model based on LRGs, constructed via multi-omics analysis and machine learning algorithms, stratified PCa patients into high-risk and low-risk groups. The high-risk group exhibited a stronger tendency for immune evasion and poorer treatment response, while the low-risk group demonstrated higher sensitivity to chemotherapeutic agents. (Wang Q. et al., 2025). Similarly, a signature comprising 10 LRGs consistently distinguished patient prognosis in validation cohorts. The high-risk group was associated with increased stromal infiltration and an immunosuppressive phenotype, along with greater sensitivity to chemotherapeutic agents such as docetaxel, whereas the low-risk group benefited more from immune checkpoint inhibitors (Du et al., 2025). Additionally, a model built with five LRGs effectively predicted disease-free survival in PCa patients. The high-risk group displayed higher tumor mutation burden and M2 macrophage infiltration, as well as increased sensitivity to drugs like docetaxel (Pan et al., 2024). These models not only provide molecular markers for prognostic assessment but also establish a foundation for developing individualized treatment strategies.

## 6 The role and mechanism of lactylation in RCC

**FIGURE 5:**
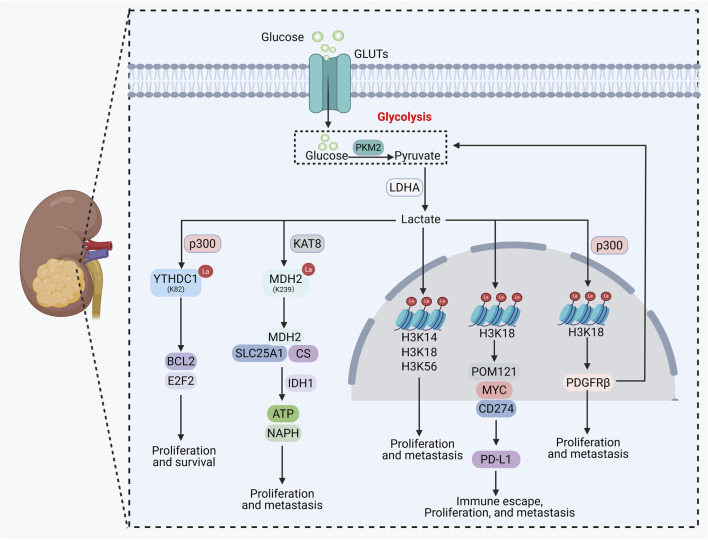
The role and mechanism of lactylation in renal cell carcinoma.

In RCC, the role of lactylation—particularly histone lactylation—is also significant (Table 1; Figure 5). VHL inactivation is a key driver event in clear cell RCC (ccRCC) (Cotta et al., 2023), which promotes enhanced glycolysis and lactate accumulation through a HIF-dependent mechanism, thereby inducing H3K18la. H3K18la recruits EP300 to activate the transcription of platelet-derived growth factor receptor β (PDGFRβ), and PDGFRβ signaling in turn further promotes glycolysis and lactate production, forming a positive feedback loop that drives tumor proliferation and metastasis (Yang et al., 2022). Similarly, in a mouse model of RCC brain metastasis, the metabolite L-2-hydroxyglutarate upregulates the transcription of HIF1α by promoting H3K18la, thereby suppressing ferroptosis in tumor cells, enhancing their proliferative and migratory capacities, and facilitating RCC brain metastasis (Liu et al., 2025g). Additionally, FKBP10 binds to LDHA and promotes its phosphorylation, enhancing LDHA activity and lactate production. This subsequently induces histone lactylation modifications such as H3K14la and H3K18la, regulating the expression of metabolism-related genes to facilitate ccRCC progression (Liu et al., 2024). Histone lactylation is also closely linked to immune evasion in RCC. Studies have shown that NSUN2 stabilizes NEO1 mRNA through an m^5^C-dependent mechanism, enhancing glycolysis and elevating H3K18la levels. The latter upregulates PD-L1 via the MYC/POM121/CD274 axis, mediating immune escape (Wang K. et al., 2025). Furthermore, in the TME, lactate accumulation resulting from VHL mutation regulates the activation of cancer-associated fibroblasts (CAF) through histone lactylation, exacerbating immunosuppression (Kong et al., 2024).

Non-histone lactylation also plays an important role in the malignant progression of RCC. On one hand, hypoxia induces p300-mediated lactylation of YTHDC1, enhancing its phase separation ability and promoting the formation of nuclear condensates. These condensates protect the mRNAs of oncogenes such as BCL2 and E2F2 from degradation by the PAXT complex, thereby facilitating RCC progression (Dai et al., 2025). On the other hand, in RCC, KAT8-catalyzed lactylation of mitochondrial malate dehydrogenase 2 (MDH2) enhances its enzymatic activity, which it elevating NADPH levels through an IDH1-dependent pathway mediated by the MDH2/CS/SLC25A1 complex, this helps tumor cells resist oxidative stress and maintain mitochondrial function, ultimately promoting the proliferation, migration, and invasion of RCC cells (Tang et al., 2025b). Although research on non-histone lactylation in renal cancer remains limited, its potential should not be overlooked.

Similarly, prognostic models constructed based on LRGs show great potential for clinical application in RCC patients. A study focusing on LRGs in CAFs identified TIMP1 as a hub gene, whose high expression is significantly associated with poor prognosis in ccRCC patients and may serve as a predictor of tumor aggressiveness (Kong et al., 2024). Furthermore, a risk model integrating LRGs and m^6^A-related genes effectively distinguished survival outcomes among ccRCC patients. The high-risk group exhibited higher tumor mutation burden and microsatellite instability, along with increased sensitivity to immunotherapy (Yang L. et al., 2023). These models offer promising directions for prognostic prediction in RCC, though further research is needed to validate their predictive value.

## 7 Conclusions and prospects

As a novel PTM linking cellular metabolism and epigenetics, lactylation has demonstrated substantial potential for clinical application in urological tumors. Current studies have established that histone lactylation regulates the transcription of key genes such as ZEB1 and PDGFRβ by remodeling chromatin structure, participating in processes like EMT in BC and proliferation and metastasis in RCC. Non-histone lactylation influences core biological processes such as DNA repair and mRNA stability by modulating protein stability, enzymatic activity, or subcellular localization, thereby mediating drug resistance and malignant progression in tumors. Meanwhile, prognostic models constructed based on LRGs demonstrate clinical value in stratifying patient risk, predicting treatment response, and estimating survival outcomes, offering potential tools for personalized diagnosis and treatment. These achievements not only elucidate the central role of lactylation in the development and progression of urological malignancies but also establish a theoretical foundation for its use as a novel therapeutic target, advancing research in the field of metabolism-epigenetics crosstalk.

However, research on lactylation requires deeper exploration of its molecular mechanisms. The regulatory roles of lactylation are highly complex: within the same disease, lactylation of different proteins can exert distinct effects, while lactylation of the same protein may lead to divergent outcomes across different diseases. Furthermore, BC, RCC, and PCa exhibit significant differences in their pathogenesis and oncogenic mechanisms. Consequently, it is challenging to conduct in-depth analysis and integration of the functions and mechanisms of lactylation across these three distinct malignancies. Future studies are needed to further unravel the intricate regulatory network of lactylation in urological tumors. First, although previous studies have found that non-histone lactylation sites far outnumber those on histones (Yang Z. et al., 2023), research in urological malignancies has predominantly focused on histone lactylation. The role of non-histone lactylation in urological malignancies remains insufficiently explored. Second, several studies suggest that crosstalk often occurs between different PTMs (Peng and Du, 2025; Qu et al., 2025), yet the interactions between lactylation and other PTMs—such as acetylation and phosphorylation—in urological malignancies are still unclear. For instance, whether competitive binding occurs between lactylation and acetylation at the histone H3K18 site. Deciphering these cross-regulatory networks is essential for understanding the specificity of lactylation-mediated regulation. Moreover, most current studies have predominantly focused on the tumor-promoting role of lactylation in urological tumors, with only limited evidence suggesting its potential tumor-suppressive functions. Future research should comprehensively investigate the dual roles of lactylation in these malignancies. Finally, it remains unclear whether the oncogenic or tumor-suppressive effects of lactylation are context-dependent. For instance, whether lactylation exerts distinct functions across different tumor types, microenvironmental conditions (e.g., hypoxia levels, lactate concentration), modification targets, and specific modification sites requires further investigation.

Challenges in clinical translation are even more pronounced. Most existing prognostic models are based on bioinformatic analyses of public databases and lack independent validation through multi-center, large-sample clinical cohorts. Their applicability across different ethnicities and clinical stages remains unclear, and the potential predictive value of these models still requires confirmation via prospective studies. Although anti-tumor strategies targeting lactylation have shown broad potential, currently validated effective drugs remain very limited, and their safety profiles require further study. Several challenges persist in this field. On one hand, existing HDAC inhibitors, LDHA inhibitors, and similar agents often lack sufficient specificity, potentially affecting normal cellular functions while suppressing tumors. There is an urgent need to develop more selective compounds and systematically evaluate their impact on healthy tissues. Utilizing targeted technologies such as nano-delivery systems and antibody-drug conjugates to achieve precise regulation of tumor cells may represent a critical approach to overcoming this bottleneck (Dong et al., 2019; Maksymova et al., 2025; Yu et al., 2024). On the other hand, optimizing combination therapy regimens is another key direction. Preclinical studies indicate that lactylation inhibitors combined with immune checkpoint blockers or chemotherapy drugs can produce significant synergistic effects (Deng J. et al., 2025; Wu et al., 2025; Liang et al., 2025). However, their mechanisms of action, dosing schedules, optimal ratios, and long-term safety profiles still need to be clarified through subsequent clinical trials.

Future research requires coordinated breakthroughs in three key areas: technological innovation, mechanistic exploration, and clinical translation. At the technological level, current methods exhibit limited accuracy in detecting low-abundance lactylation, particularly on non-histone proteins. There is an urgent need to develop high-resolution detection tools that combine ultra-sensitive mass spectrometry with highly specific antibodies to improve the precision of lactylation level quantification. Furthermore, integrating lactylomics with metabolomic and transcriptomic data will help construct “metabolism-modification-phenotype” association networks, enabling the characterization of lactylation features in cisplatin-resistant subpopulations in BC and neuroendocrine-differentiated cells in PCa. Mechanistically, future studies should focus on elucidating the spatiotemporal dynamics of lactylation to determine whether temporal or spatial variations in lactylation patterns occur in urological malignancies. Additionally, applying structural biology approaches to decipher the competitive or cooperative relationships between lactylation and other PTMs will significantly deepen our understanding of lactylation’s role in urological malignancies. In terms of clinical translation, multi-dimensional prognostic models must be optimized by incorporating lactylation levels, gene expression profiles, and clinicopathological features to enhance predictive accuracy. Moreover, developing highly selective inhibitors targeting key lactylation-related enzymes or proteins will be essential to minimize off-target effects and improve therapeutic specificity.

In summary, significant progress has been made in understanding the role of lactylation in urological malignancies, yet the complexity of its regulatory mechanisms and the challenges in clinical translation require continued exploration. With advancements in technological methods and in-depth mechanistic studies, lactylation is expected to become a novel breakthrough for precision diagnosis and treatment in urological malignancies, providing a solid theoretical foundation and practical basis for improving patient prognosis.
